# Gentle touch perception: From early childhood to adolescence

**DOI:** 10.1016/j.dcn.2017.07.009

**Published:** 2017-08-18

**Authors:** Ilona Croy, Isac Sehlstedt, Helena Backlund Wasling, Rochelle Ackerley, Håkan Olausson

**Affiliations:** a Department of Psychotherapy and Psychosomatic Medicine, University Hospital Dresden, Technische Universität Dresden, Fetscherstr. 74, 01307 Dresden, Germany; b Center for Social and Affective Neuroscience, Linköping University, Linköping, Sweden; c Department of Psychology, University of Gothenburg, Box 500, SE 40530, Gothenburg, Sweden; d Department of Physiology, University of Gothenburg, Box 432, SE 40530, Gothenburg, Sweden; e Laboratoire de Neurosciences Intégratives et Adaptatives (UMR 7260), Aix-Marseille Université − CNRS, 13331 Marseille CEDEX 03, France

**Keywords:** Children, Touch, C-tactile, Affective, Psychophysical, Somatosensory

## Abstract

Affective touch plays an important role in children’s social interaction and is involved in shaping the development of the social brain. The positive affective component of touch is thought to be conveyed via a group of unmyelinated, low-threshold mechanoreceptive afferents, known as C-tactile fibers that are optimally activated by gentle, slow, stroking touch. Touch targeting these C-tactile fibers has been shown to decrease the heart rate in infants. The current study investigated the relationship between age and psychophysical ratings in response to affective touch. A total of n = 43 participants (early childhood: aged 5–8 years, 9 girls, 12 boys; late childhood: aged 9–12 years, 12 girls, 10 boys) were presented with C-tactile optimal and sub-optimal stroking velocities and rated touch pleasantness on an affective pictorial scale. For both age groups, we found that children preferred C-tactile-targeted stimulation. A comparison with previously published data showed that the children’s preference for C-tactile-targeted stimulation was similar to those obtained in adolescents and adults. We speculate that the effect of C-tactile-targeted touch, which is linked with pleasantness, shapes the children’s preference for C-tactile over non-C-tactile-targeted stimulation, and that C-tactile afferent stimulation is important for social development.

## 1 Introduction

Interpersonal, affective touch plays an important role in social interactions and has beneficial health implications (McGlone et al., 2014). Children, especially, seek such stimulation and benefit from interpersonal touch in their emotional and behavioral development (Field, 2002). Conversely, the neglect of positive touch interactions, such as in certain orphanages, results in adverse emotional, behavioral, and even physical development of the child (Spitz, 1945).

Neurophysiological studies show that positive affective components of touch are conveyed via a group of unmyelinated, low-threshold mechanoreceptive afferents, referred to as C-tactile fibers (cf. (McGlone et al., 2014) for review). These fibers respond to innocuous, mechanical stimuli (Nordin, 1990, Wessberg et al., 2003, Vallbo et al., 1999) and are most robustly activated by light stroking stimulation (Ackerley et al., 2014a; Löken et al., 2009). Further, C-tactile firing frequency is highest for touch at skin-like temperatures, suggesting that C-tactile fibers are tuned to human touch (Ackerley et al., 2014a). Intermediate velocities of stroking (1–10 cm/s) activate C-tactile fibers optimally (through increased firing frequency) and result in high pleasantness ratings; other velocities (0.1, 0.3, and 30 cm/s) however result in non-optimal C-tactile fiber activation, and lower ratings of touch pleasantness. The mean firing frequency of C-tactile fibers correlates strongly with ratings of pleasantness (Ackerley et al., 2014a; Löken et al., 2009). This is not the case for myelinated, fast-conducting A-beta afferents (Ackerley et al., 2014a; Löken et al., 2009), which convey discriminative touch information necessary to identify objects and for precise motor control (Johansson and Westling, 1987). Related psychophysical studies have repeatedly shown higher pleasantness ratings during stroking touch from 1 to 10 cm/s compared to slower or faster stroking velocities in adults (e.g. Ackerley et al., 2014b, Sehlstedt et al., 2016, Essick et al., 2010, Jönsson et al., 2015).

Hence, we define touch with a stroking velocity between 1 and 10 cm/s as targeted at C-tactile afferents. We previously introduced a measurement to capture the individual preference of C-tactile targeted touch (Croy et al., 2016a). We defined this index as the individual difference between the pleasantness ratings towards C-tactile targeted (3 cm/s) versus non-C-tactile targeted touch (30 cm/s), weighted by the overall touch pleasantness. This measures how much a person prefers C-tactile targeted touch over non-C-tactile targeted touch. Presently, we used this affective touch index to investigate the sensitivity to positive affective touch, which is often used in conspecific interaction and may provide insights into social cognitive deficits. Few studies have investigated such affective touch in children and it is of interest to understand how affective touch is perceived throughout childhood, during typical development and in those that have or go on to develop somatosensory deficits.

C-tactile-targeted affective touch activates the insular cortex (Morrison, 2016, Croy et al., 2016c, Gordon et al., 2013, McGlone et al., 2012, Olausson et al., 2002, Sailer et al., 2016), the orbitofrontal cortex (McGlone et al., 2012), the superior temporal sulcus (Gordon et al., 2013, Davidovic et al., 2016), and anterior cingulate cortex, but seems to bypass primary somatosensory cortex (Case et al., 2016, Olausson et al., 2008). The insula and the anterior cingulate cortex have been identified as critical for salience detection (Bressler and Menon, 2010), the superior temporal sulcus plays a crucial role in emotional processing and social cognition (Allison et al., 2000), and the orbitofrontal cortex is involved in reward perception. The observation that touch is sought after and experienced as pleasant, even for extended periods of time (Triscoli et al., 2014), may be explained by the recruitment of striatal reward areas during extended periods of stroking (Sailer et al., 2016). In five-year-old children, the frequency of maternal touch predicts resting state activity and connectivity in the superior temporal sulcus and temporo-parietal junction − regions which are highly involved in the social brain network (Brauer et al., 2016). Similarly, children diagnosed with autism show decreased brain responses to C-tactile-targeted touch in the insula, superior temporal sulcus, and temporo-parietal junction (Kaiser et al., 2016).

Touch forms an important part of our development that starts in utero and continues from birth. Pre-term infants who receive human touch have more advanced social development than those who do not ((Kramer et al., 1975); see also (Pepino and Mezzacappa, 2015) for a review). There are few studies that have investigated positive affective touch in childhood and these have focused on the physiological effects (e.g. cortical processing: (Brauer et al., 2016, Kida and Shinohara, 2013, Bjornsdotter et al., 2014; heart rate: Fairhurst et al., 2014). Yet, this is a time when children are developing a sense of self and identity, both personally and socially, and form perceptions about touch. We have recently shown that the preference of C-tactile optimal stimulation (i.e. slow stroking) over C-tactile sub-optimal stimulation (very slow or fast stroking) is maintained across the lifespan (13–82 years) in a cross-sectional study (Sehlstedt et al., 2016). Based on the premise that C-tactile fiber input is rewarding and involved in shaping the social brain, we hypothesize that children should prefer tactile stimulation that targets C-tactile fibers. That is, children should prefer stroking applied with a C-tactile optimal stroking velocity of 3 cm/s over stroking applied with faster or slower velocities.

## 2 Material and methods

### 2.1 Participants

The investigation conformed to local ethical approval and was performed in accordance with the Declaration of Helsinki. A total of 43 healthy Swedish children were included. The participants were divided into two groups: early childhood (aged 5–8 years, mean 6.4 years, ±1.1 SD, 9 girls, 12 boys) and late childhood (aged 9–12 years, mean 10.4 years ±1.1 SD, 12 girls, 10 boys). Exclusion criteria were sensory impairments, severe neurological disorders, and diabetes mellitus. All children were in good health, as reported by their parents. Body mass index of each child was within the respective age norm.

For comparison, previously published data (Sehlstedt et al., 2016) from an adolescent group of participants (aged 13–18 years, mean 15.5 years, ±1.5, 20 males, 20 females), an adult group (aged 19–44 years, mean 31.7 years, ±7.9, 20 males, 21 females), and a late adulthood group (aged 45–82 years, mean 60.1 years, ±10.0y SD, 20 males, 19 females) were reanalyzed.

### 2.2 Experimental set-up

Similar to our previous study on the development of touch in adolescents and adults (Sehlstedt et al., 2016), participants were seated in an upright hospital bed in a well-ventilated room and were presented with tactile stimuli as detailed below. Participants were prevented from seeing the touch stimuli by the use of shielding glasses.

### 2.3 Touch stimuli

The touch stimuli were delivered using a rotary tactile stimulator (Dancer Design; Wirral, UK) that provides high-precision brush strokes at a calibrated force of 0.4 N. Participants were stroked on their dorsal left forearm (palm facing down) in a proximal-to-distal direction. Brush strokes were given with a flat, soft brush made out of goat hair that traversed approximately 6 cm of skin at three different velocities (0.3, 3, and 30 cm/s) presented in a pseudo-randomized order. Each stroking velocity was presented 3 times; however, a velocity was not repeated until all velocities had been presented an equal amount of times. Further, randomization order was counterbalanced across the participants. The inter-trial interval was set to 25 s from the end of one brush stroke to the start of the next.

After each brush stroke, the children were asked to point to a pictogram on an affective ratings scale (smiley faces), which described best how the stimulation felt (“Can you show me how this touch feels?”). The principal procedure was similar to paradigms used in previous studies (e.g. Löken et al., 2009, Ackerley et al., 2014b, Sehlstedt et al., 2016, Essick et al., 2010, Jönsson et al., 2015), however the rating scale was adapted for children (Chaplin et al., 2008, Jäger, 2004). In contrast to previous studies, touch stimuli were not rated using visual analog scales (VAS), but rather an affective pictorial scale with five levels (Jäger, 2004). Such a scale has been validated to use in children as young as 4 years old and has provided reliable and comparable results to other perceptual scales, yet is simple to use and easy for them to understand, even for children with disabilities (Chaplin et al., 2008). A pilot study by ourselves also revealed that the younger children found the affective pictorial scale easier to understand and less abstract than the VAS. After each touch stimulation, the children were instructed to point to the smiley face that depicted “how this touch made them feel.” Before the experiment started, we always explained the scale to the children. We furthermore performed three ratings of the pleasantness of stroking touch before starting the experiment, to ensure that they understood the task.

### 2.4 Statistical analyses

Statistical comparisons were made using SPSS (version 22; Armonk, NY: IBM Corp). All statistical tests were performed using non-parametric tests due to the non-linear, ordinal scaling of the affective pictorial scales and the skewed distribution of results.

The ratings from the affective pictorial scales were converted into numbers (1-very bad, 2-bad, 3-neutral, 4-happy, 5-very happy) and the median of the three ratings per stroking velocity was calculated.

In order to examine the main effect of age and stroking velocity, as well as potential interactions of both, we used generalized estimating equations (GEE) with an ordinal logistic response model. An ordinal model was used, as metric characteristics (especially equidistance) cannot be assumed for the dependent variable (median of affective pictorial scale ratings) and as data was skewed to the right (to high values of pleasantness). Stroking velocity (3 levels) served as a within-subject factor and age group (2 levels) and sex (2 levels) as between-subject factors. Sex was included as a between-subject factor because previous research reports mixed results of whether female participants rate C-tactile stimuli more pleasant than male participants do (Croy et al., 2014; Jönsson et al., 2017), or whether there are no significant gender differences (Sehlstedt et al., 2016). Main effects and all two-way interaction effects were examined. The randomization order of touch presentation was included in the model as covariate in order to adjust for potential effects of randomization order, which may occur in the rather small sample size. Effect sizes are given using Cohen’s d. Post-hoc comparisons between velocities were performed using Wilcoxon Signed Ranks tests for non-parametric data and were Bonferroni-corrected by a factor 3 to adjust for multiple comparisons in three analyses of velocity. This corrected value is indicated by “p_corr_”. We furthermore repeated the whole GEE analysis as described above using age as a continuous instead of a categorical measurement. This was done in order to fully use the statistical power of the rather small data set.

For individual comparisons, the ‘affective touch index’ was computed for each participant. The affective touch index refers to the specific preference for C-tactile-targeted touch and represents the extent to which an individual prefers the C-tactile optimal stroking velocity of 3 cm/s to the C-tactile suboptimal velocity of 30 cm/s. For the suboptimal velocity we used 30 cm/s because this is a better control than 0.3 cm/s. Previous behavioral studies have shown equivalence in the rated touch intensity between stroking at 3 cm/s and 30 cm/s, whereas stroking at 0.3 cm/s is felt as less intense (Sehlstedt et al., 2016; Jönsson et al., 2017). Also, microneurography studies have shown that at slower velocities (e.g. 0.3, 0.1 cm/s), there is plenty of C-tactile activity, albeit at a lower frequency, whereas little C-tactile is found during stroking at 30 cm/s (Löken et al., 2009, Ackerley et al., 2014a). The index is computed as follows (compare (Croy et al., 2016a)):

$$\text{affective}\;\text{touch}\;\text{index}=\frac{\left(\text{Pleasantness}\;\;\;3\frac{\text{cm}}{\text{s}}-\text{Pleasantness}\;\;\;30\frac{\text{cm}}{\text{s}}\right)}{\left({\sum^{\;}}^{\;}\left(\text{Pleasantness}\;\;\;0.3\frac{\text{cm}}{\text{s}},\;\;\text{Pleasantness}\;3\frac{\text{cm}}{\text{s}},\;\;\text{Pleasantness}\;\;3\frac{\text{cm}}{\text{s}}\right)/3\right)}$$

Hence, a positive affective touch index indicates that an individual prefers stroking at 3 cm/s to stroking at 30 cm/s. The affective touch index was then correlated with age using Spearman’s correlation coefficient.

The percentage of individuals with a positive, negative, and neutral affective touch index was calculated in each group and compared using the Chi^2^ test. Specific analyses were carried out in order to compare the current data to previously published data from adolescents and adults (Sehlstedt et al., 2016). These previously published data followed a very similar protocol; however, pleasantness ratings were collected using a VAS with a range of −10 to 10. Since the range and anchors of the scale in our current study did not match the previous study, direct comparison is problematic. To address this issue, the averaged pleasantness ratings per velocity from the previously published dataset were scaled down to fit within the same range as the affective pictorial scale by binning pleasantness ratings into 5 bins (10 to 6 = 5, 5.9 to 2 = 4, 1.9 to −2 = 3, −2.1 to −6 = 2, and −6.1 to −10 = 1). Afterwards, the affective touch index was computed as described above and each individual was grouped according to a positive, negative, or neutral affective touch index. A positive value was defined as values for C-tactile optimal touch being more than those for C-tactile suboptimal touch, a neutral value was a value of exactly zero, and a negative value was where C-tactile optimal ratings were less than C-tactile suboptimal ratings. Chi^2^ tests were used to compare the distribution of the affective touch index between the age groups.

## 3 Results

**Fig. 1:**
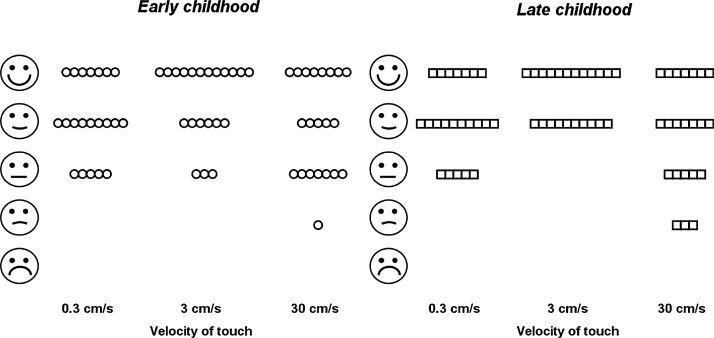
Pleasantness ratings following soft stroking on the forearm with the C-tactile suboptimal stroking velocity of 0.3 and 30 cm/s and the optimal stroking velocity of 3 cm/s for early and late childhood. The highest density of positive ratings is observed for the 3 cm/s velocity of touch. Ratings were done by young (5–8 years, N = 21) and older (9–12 years, N = 22) children. Each circle represents the median rating of a single child. Children in both age groups significantly preferred the C-tactile optimal over the suboptimal velocities.

There was a significant main effect of stroking velocity on pleasantness ratings (Wald Chi^2^ = 12.9, p = 0.002, d = 1.3, compare Fig. 1), with 3 cm/s being rated significantly more pleasant than 0.3 cm/s (Z = 2.8, p_corr_ = 0.015, d = 0.57) and 30 cm/s (Z = 3.3, p_corr_ = 0.003, d = 0.73), and no significant difference between pleasantness ratings of 0.3 and 30 cm/s (Z = 1.4, p_corr_ = 0.49, d = 0.23).

There was no significant main effect of age group (Wald Chi^2^ = 0.02, p = 0.88) or sex (Wald Chi^2^ = 0.003, p = 0.96) on pleasantness ratings. Further, there was no significant age by velocity interaction (Wald Chi^2^ = 0.45, p = 0.80) and no significant sex by velocity interaction (Wald Chi^2^ = 2.3, p = 0.32), indicating that neither the overall pleasantness ratings nor the stroking velocity-dependent pleasantness ratings changed as a function of sex or age. Inclusion of stimulus presentation order as a covariate did not change the results (main effect of stroking velocity: Wald Chi^2^ = 12.96, p = 0.002; no other significant main or interaction effects).

**Fig. 2:**
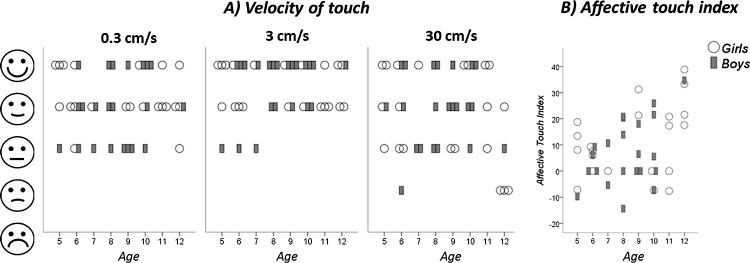
Individual values for age effects on affective touch. A) Pleasantness ratings following soft stroking on the forearm with the C-tactile suboptimal stroking velocity of 0.3 and 30 cm/s and the optimal stroking velocity of 3 cm/s are presented for each age separately. B) The affective touch index shows a positive correlation with age (note that identical values are presented above each other). Older children prefer C-tactile targeted touch over non-c-tactile targeted touch.

Using age as a continuous variable, instead of age groups, the results changed slightly. The significant main effect of stroking velocity on pleasantness ratings remained robust (Wald Chi^2^ = 13.2, p = 0.001, d = 1.4), but further significant main effects were found for age (Wald Chi^2^ = 15.6, p = 0.029) and sex (Wald Chi^2^ = 5.4, p = 0.020) and there was a significant age by sex interaction effect (Wald Chi^2^ = 41.9, p < 0.001). However, there was no significant age (or sex) by velocity interaction. The age effect indicated that older children rated touch as more pleasant than younger children; the sex effect showed that girls rated touch as more pleasant than boys. The interaction showed that boys increased in their pleasantness ratings with increasing age, while the girls’ ratings increased to a lesser extent (compare Fig. 2a). This was especially found for the ratings for the C-tactile optimal velocity (3 cm/s) and a positive correlation between the affective touch index and age was found (r = 0.34, p = 0.028; compare Fig. 2b).

On an individual level, 48% of the early childhood group and 59% of the late childhood group demonstrated a positive affective touch index, meaning they preferred the C-tactile optimal stroking of 3 cm/s over the suboptimal stroking velocity of 30 cm/s. Only 14% (early childhood) and 18% (late childhood) demonstrated a negative affective touch index and rated the C-tactile suboptimal stimuli more pleasant than the optimal stimulus. Thirty-eight percent (early childhood) and 23% (late childhood) did not differentiate between stroking velocities. In 30% of the cases, this lack of preference was explained by ceiling effects, meaning these children rated every type of touch in the highest category.

**Fig. 3:**
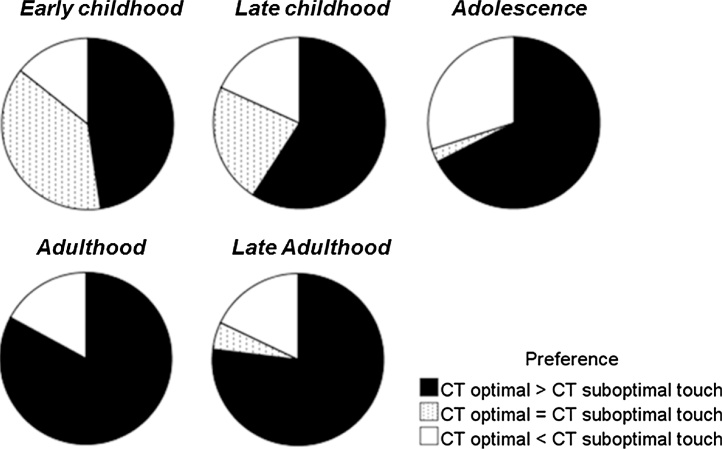
Preference of C-tactile optimal (3 cm/s) compared to C-tactile suboptimal stroking across age groups (affective touch index). Data from the present paper are aggregated with data from (Sehlstedt et al., 2016) for visualization purpose. The proportion of individuals in the respective age group that preferred stroking with 3 cm/s over stroking with 30 cm/s is indicated in black. There were no significant differences between the age groups in velocity preference.

These data were compared to data from adolescents and adults (Sehlstedt et al., 2016), which were reanalyzed for this purpose. In all age groups, the C-tactile optimal stimuli were preferred by more individuals than C-tactile suboptimal stimuli were (Fig. 3). Preferences differed significantly between groups (Chi^2^ = 33.6, p < 0.001); however this effect was based on the higher amount of indifferent ratings (ratings to C-tactile optimal touch = ratings to C-tactile suboptimal touch) in the childhood groups. There were no significant differences in the proportion of people with a negative affective touch index across age groups (Chi^2^ = 3.3, p = 0.52).

## 4 Discussion

We find that children preferred C-tactile-targeted stroking on the forearm, as compared to stroking applied with faster or slower velocities. As a whole, 86% of the children in the younger age group (5–8 years), and 82% of the children in the older age group (9–12 years) rated C-tactile targeted stroking as equally pleasant or more pleasant than non-C-tactile targeted touch. With increasing age, the affective touch index increased. Hence, the older the children, the more they preferred C-tactile optimal over C-tactile suboptimal stroking.

We show that C-tactile-targeted stimulation (i.e. gentle, slow stroking of the hairy skin) is an important facet of affective touch from childhood. Previous studies have shown that C-tactile touch reduces the heart rate of adults (Pawling et al., 2017, Triscoli et al., 2017) and infants (Fairhurst et al., 2014). Gentle touch in general (i.e. to glabrous skin and not just the C-tactile afferent innervated hairy skin) activates higher cortical areas, associated with the processing of emotion (e.g. perceived pleasantness), as found both in infants (Kida and Shinohara, 2013) and in adults (McGlone et al., 2012). These studies show the complexity in processing pleasant touch and its implications. Taken together, these data indicate that gentle touch processing is present in infants and evokes specific central and autonomic responses.

The effects of optimal C-tactile fiber stimulation seem to be advantageous. It is linked with pleasantness (Löken et al., 2009, Ackerley et al., 2014a) and it reduces heart rate in infants (Fairhurst et al., 2014). Gentle stroking also has beneficial health effects in preterm babies (Kramer et al., 1975, Pepino and Mezzacappa, 2015, Field et al., 2010). From the perspective of the “toucher”, a recent study shows that parents stroke their babies using slow velocities, optimal for targeting C-tactile fibers (Croy et al., 2016b). This suggests that infants are familiar with such touch and the familiarity of C-tactile targeted touch may shape a child’s preference for C-tactile- over non-C-tactile-targeted stimulation.

Combining these results with our previous study (Sehlstedt et al., 2016) leads us to conclude that humans prefer C-tactile-targeted touch over non-C-tactile-targeted touch from childhood to late adulthood. Differences between age groups depended on those participants who rated C-tactile-targeted touch as high as non-C-tactile-targeted touch. We assume that this difference is explained by the use of different scales for children and adolescents/adults. The finer tuned VAS scales, used with adolescents and adults, allows for more precise ratings compared to the affective pictorial scales. However, one may also suspect that touch in general is highly pleasurable early in development and that subsequently pleasure from non-C-tactile touch declines.

We find a proportion of individuals throughout all age groups who preferred non-C-tactile-targeted touch. There may be multiple contributing factors, such as inter-personal preferences, expectations, and experience, among others. Such data are inherently noisy, due to individual differences, but our work and previous studies have shown increased pleasantness for skin stroking around 3 cm/s (e.g. (Ackerley et al., 2014a; Löken et al., 2009; Ackerley et al., 2014b; Sehlstedt et al., 2016). This is a general finding, although it only appears in group data. Individuals have their own specific preferences; hence the findings from one person cannot be used to diagnose disorders. However, comparisons may be made over time, as it has been recently shown, that an individual’s preference for pleasant touch is rather stable over a time interval of two weeks (Luong et al., 2017). This hints towards the notion, that touch pleasantness is, at least partly, determined by stable factors and may be used prognostically in individuals.

C-tactile-targeted touch has also been linked to social behavior deficits. The perception and cortical processing of C-tactile targeted-stimuli is reduced in autistic children (Kaiser et al., 2016) as well as in adults with high levels of autistic traits (Voos et al., 2013), compared to the respective age–matched, healthy controls. Further, adults with autism are sensitive to certain aspects of touch (Cascio et al., 2008), and individuals with high levels of autistic traits show a reduced affective touch index (Croy et al., 2016a). Likewise, the experimental reduction of peripheral tactile sensitivity in mice, which was caused by the deletion of specific genes in somatosensory neurons, leads to reduced social interaction in those animals (Orefice et al., 2016). Hence, it is possible that C-tactile perception constitutes a stable trait in humans that is related to social behavior, indicating that humans with a high affective touch index (or C-tactile perception) are more interested in or responsive to social interactions.

Our present data were obtained in a controlled laboratory situation, with ordinal data, which increases the internal validity of our results, but at the same time limits the ecological validity. A previous study showed equivalence between pleasantness ratings stroking with a robot and with a hand-held brush (Triscoli et al., 2013). More naturalistic studies are warranted about the interactions in human touch, given that higher-order processes (e.g. perception about the person delivering touch; (Gazzola et al., 2012)) can affect how touch is processed. To this end, the use of different touch stimuli (e.g. more or less pleasant contact surfaces) may aid in understanding how pleasantness is derived. Further investigations may also investigate the use of our touch measures for prognostic applications (e.g. the affective touch index), but further research is needed. It is not clear yet whether C-tactile perception remains constant over the course of several years. It is a challenging future question whether individuals who do not prefer C-tactile touch as children keep this non-preference through the course of life.

## Funding source

This study was supported by a grant from the Marcus och Amalia Wallenbergs Minnesfond to IC (MAW 2014.0009).

## Conflict of Interest

None.
